# Thoracoscopic surgical case of an ectopic mediastinal parathyroid adenoma detected by chance: a case report

**DOI:** 10.1186/s12893-019-0641-2

**Published:** 2019-11-14

**Authors:** Shota Mitsuboshi, Hideyuki Maeda, Hiroe Aoshima, Tamami Isaka, Takako Matsumoto, Hiromi Onizuka, Masato Kanzaki

**Affiliations:** 1 Department of Thoracic Surgery, Tokyo Women’s Medical University, 8-1 Kawada-cho, Shinjuku-ku, Tokyo, 162-8666 Japan; 2 Department of Surgical Pathology, Tokyo Women’s Medical University, 8-1 Kawada-cho, Shinjuku-ku, Tokyo, 162-8666 Japan

**Keywords:** Video-assisted thoracoscopic surgery, Ectopic mediastinal parathyroid tumor, Technetium-99 m methoxyisobutyl isonitrile scintigraphy

## Abstract

**Background:**

Ectopic mediastinal parathyroid tumor (EMPT) is a rare cause of primary hyperparathyroidism (PHPT); it is difficult to resect using the cervical approach. We describe a case of using video-assisted thoracic surgery (VATS) for EMPT resection.

**Case presentation:**

A 67-year-old woman with a history of postoperative thyroid cancer had no symptoms. She was diagnosed with PHPT and underwent thyroid cancer surgery. She had serum calcium and intact parathyroid hormone (PTH) levels of 11.1 mg/dL and 206 pg/mL, respectively. Chest computed tomography showed a 10-mm nodule in the anterior mediastinum. Technetium-99 m methoxyisobutyl isonitrile scintigraphy showed an abnormal uptake lesion in the anterior mediastinum. She was diagnosed with PHPT caused by EMPT and underwent VATS. The pathological examination confirmed parathyroid adenoma. Her serum calcium and intact PTH levels were normal from 15 min after tumor resection. She has had no recurrence of EMPT.

**Conclusions:**

The VATS approach was effective for the resection of EMPT.

## Background

Primary hyperparathyroidism (PHPT) is a disease that excessively secretes parathyroid hormone (PTH) and causes osteoporosis, ureteral stone, and hypercalcemia. The incidence of asymptomatic PHPT that is discovered by chance during examination of other diseases has been increasing. Ectopic mediastinal parathyroid tumor (EMPT) is a rare cause of PHPT. Although most parathyroid adenomas are located in the neck, only 1–2% of them are located in the mediastinum [1]. As surgical resection of EMPT is often difficult to perform using the cervical approach, thoracoscopic surgical resection has been used for the resection. We describe a case of video-assisted thoracoscopic surgery (VATS) for excision of EMPT. In this case, detailed preoperative information and magnified visual fields through the thoracoscope led to the correct tumor location.

## Case presentation

**Fig. 1:**
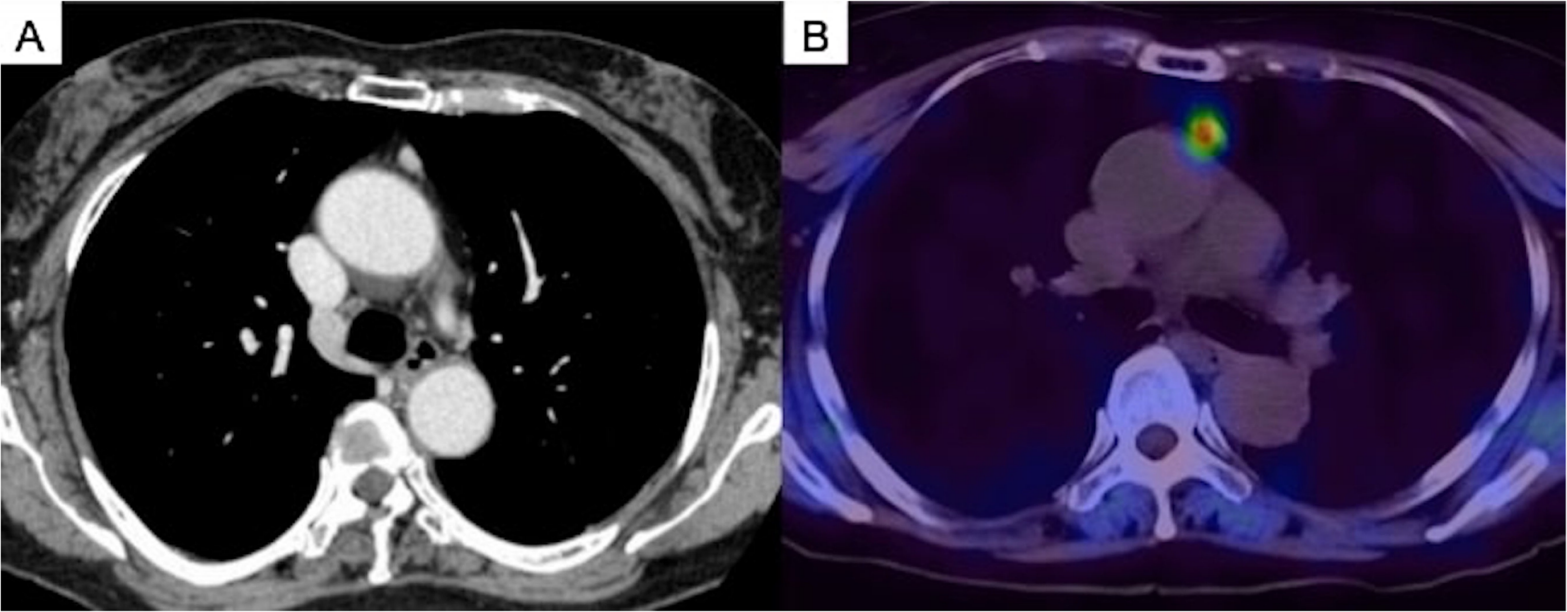
Imaging findings. (**a**) Chest computed tomography scan showing a 10-mm nodule in the anterior mediastinum. (**b**) Technetium-99 m methoxyisobutyl isonitrile scintigram showing an abnormal uptake lesion in the anterior mediastinum

A 67-year-old woman with a history of thyroid cancer surgery had no symptoms. Her serum calcium and intact PTH levels were high according to the blood biochemical examination when she underwent thyroid cancer surgery. Chest computed tomography showed a 10-mm nodule in the anterior mediastinum (Fig. 1a). Technetium-99 m methoxyisobutyl isonitrile (^99m^Tc-MIBI) scintigraphy showed an abnormal uptake lesion in the anterior mediastinum (Fig. 1b). She was diagnosed with PHPT caused by EMPT. Although she was followed up for 7 years, her serum calcium and PTH levels increased. Because the cervical approach was considered difficult for surgical resection, she was referred to our department. Preoperatively, she had a serum calcium level of 11.1 mg/dL (normal range, 8.5–9.9 mg/dL) and intact PTH level of 206 pg/mL (normal range, 16–65 pg/mL).

**Fig. 2:**
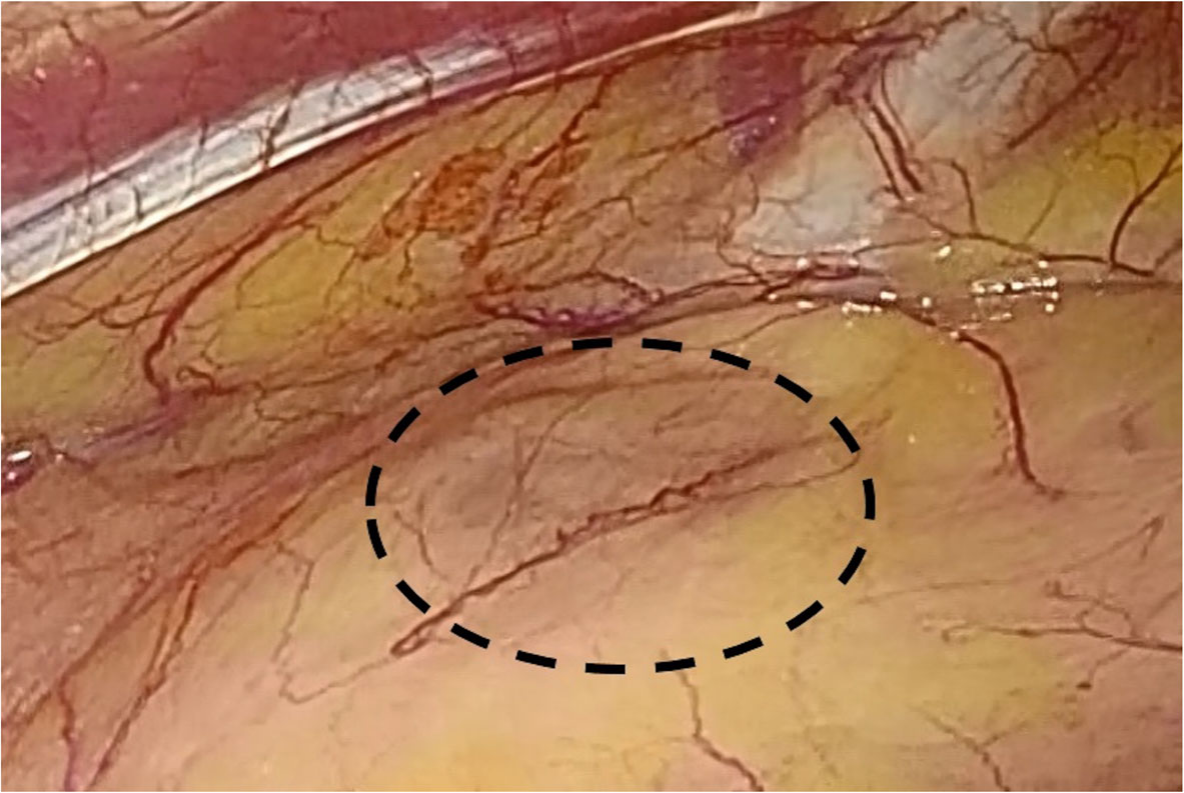
Intraoperative findings. A dark red tumor is observed in the anterior mediastinum (dotted circle)

Under general anesthesia, she was intubated with a double-lumen endotracheal tube and positioned in a right lateral decubitus position. A 4-cm vertical skin incision along the left breast trailing edge at the fourth rib was made, mini-thoracotomy was performed at the fourth intercostal space, and an XXS size wound retractor (Alexis® Wound Retractor; Applied Medical, Rancho Santa Margarita, CA, USA) was placed. A 10.5-mm thoracoport was inserted at the sixth intercostal space of the anterior axillary line and a rigid 30° thoracoscope was inserted into the seventh intercostal space of the middle axillary line. By careful inspection of the anterior mediastinum using a thoracoscope, we found a small 15-mm-diameter dark red nodule, which differed in color from its surroundings, inside the thymus and in front of the ascending aorta (Fig. 2). Without fracturing its capsule, the tumor was removed with thymic tissue. Fifteen minutes after resecting the nodule, her serum PTH level decreased immediately and became 27 pg/mL.

**Fig. 3:**
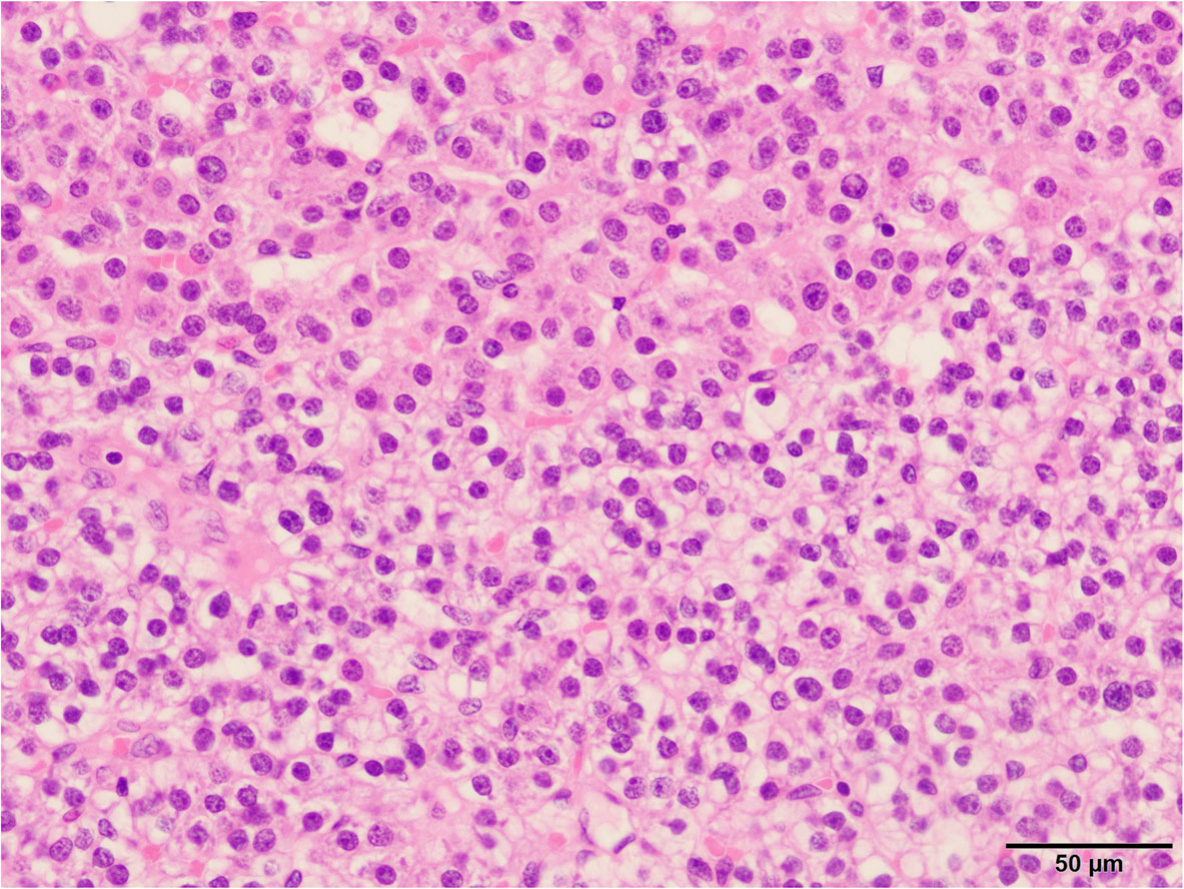
Pathological findings. Macroscopic views of the tumor confirmed the diagnosis of an ectopic mediastinal parathyroid tumor. The specimen is stained with hematoxylin-eosin. The tumor consists of chief and eosinophilic cells

The pathological examination confirmed the diagnosis of parathyroid adenoma (Fig. 3). She had a serum calcium level of 8.9 mg/dL on the first postoperative day, and her postoperative course was uncomplicated. She has had no increase of serum calcium and PTH levels and no recurrence of EMPT.

## Discussion and conclusions

PHPT causes hypercalcemia, urolithiasis, and osteoporosis due to hypersecretion of PTH. In PHPT, the incidence of EMPT is rare at about 1–2% [1]. The superior parathyroid glands develop from the fourth pharyngeal sac and descend along the esophagus with the thyroid gland. Therefore, parathyroid glands may be present in the posterior mediastinum. As the inferior parathyroid glands develop from the third pharyngeal sac and descend with the thymus, the parathyroid gland may be present in the anterior mediastinum [2]. In this case, the parathyroid adenoma was found in the anterior mediastinum, so it was considered to be EMPT derived from the inferior parathyroid gland.

Recently, the incidence of asymptomatic PHPT has increased because of improvement of examinations, which has resulted in an increase in surgical cases. The National Institutes of Health criteria for surgery in asymptomatic PHPT are as follows: (i) serum calcium level > 1.0 mg/dl above the normal upper limit; (ii) creatinine clearance < 60 ml/min, the presence of nephrolithiasis or nephrocalcinosis, or marked hypercalciuria (> 400 mg/dl); (iii) bone density T-score < − 2.5 standard deviation or medical history of vulnerable fracture; and (iv) age < 50 years. When patients meet one of these criteria, surgery is recommend [3]. In the present case, the serum calcium level increased by more 1.0 mg/dl above the normal upper limit.

PHPT is diagnosed based on serological and imaging findings. Serologically, PHPT shows high serum calcium and PTH levels and low serum phosphorus levels. For the treatment of EMPT, localization diagnosis by imaging examinations, such as computed tomography, magnetic resonance imaging, and ultrasonography, is important. The diagnostic rate of responsible lesions increased for PHPT using ^99m^Tc-MIBI scintigraphy. By using the intraoperative intact PTH rapid measurement in EMPT surgery, the failure rate of surgery decreased from 21.2 to 3% [4]. Further, when using the preoperative diagnosis and intraoperative intact PTH rapid measurement, a minimally invasive approach is mainly used to remove only the responsible lesion. Depending on the tumor location, various approaches have been used for EMPT surgery. If the tumor is on the head side of the brachiocephalic vein, the cervical approach is used, and if the tumor is on the foot side of the brachiocephalic vein, median sternotomy or thoracotomy is selected. Recently, minimally invasive thoracoscopic surgery has been performed for PHPT [5]. It is important to use examinations such as preoperative ^99m^Tc-MIBI scintigraphy and the intraoperative intact PTH rapid measurement to ensure tumor removal by thoracoscopic surgery.

As it is often difficult to identify EMPT intraoperatively, a method for identifying the tumor by administering methylene blue at the time of intraoperative has been reported. Injury of the capsule of the tumor causes seeding, so an easy method, such as administering methylene blue, is needed to identify the tumor [6].

For surgical treatment of EMPT, it is important to combine examinations such as preoperative ^99m^Tc-MIBI scintigraphy and the intraoperative intact PTH rapid measurement to ensure tumor removal by thoracoscopic surgery.

## Data Availability

All the data and materials supporting our findings are included within the article.

## Abbreviations

^99m^Tc-MIBI: Technetium-99 m methoxyisobutyl isonitrile
EMPT: Ectopic mediastinal parathyroid tumor
PHPT: Primary hyperparathyroidism
PTH: Parathyroid hormone
VATS: Video-assisted thoracoscopic surgery
